# Serum Response Factor Regulates Immediate Early Host Gene Expression in *Toxoplasma gondii*-Infected Host Cells

**DOI:** 10.1371/journal.pone.0018335

**Published:** 2011-03-29

**Authors:** Mandi Wiley, Crystal Teygong, Eric Phelps, Jay Radke, Ira J. Blader

**Affiliations:** 1 Department of Microbiology and Immunology, University of Oklahoma Health Sciences Center, Oklahoma City, Oklahoma, United States of America; 2 Department of Veterinary Molecular Biology & the Center for Immunotherapies to Zoonotic Diseases, Montana State University, Bozeman, Montana, United States of America; Weill Cornell Medical College, United States of Amercia

## Abstract

*Toxoplasma gondii* is a wide spread pathogen that can cause severe and even fatal disease in fetuses and immune-compromised hosts. As an obligate intracellular parasite, *Toxoplasma* must alter the environment of its host cell in order to establish its replicative niche. This is accomplished, in part, by secretion of factors into the host cell that act to modulate processes such as transcription. Previous studies demonstrated that genes encoding transcription factors such as c-jun, junB, EGR1, and EGR2 were amongst the host genes that were the most rapidly upregulated following infection. In cells stimulated with growth factors, these genes are regulated by a transcription factor named Serum Response Factor. Serum Response Factor is a ubiquitously expressed DNA binding protein that regulates growth and actin cytoskeleton genes via MAP kinase or actin cytoskeletal signaling, respectively. Here, we report that *Toxoplasma* infection leads to the rapid activation of Serum Response Factor. Serum Response Factor activation is a *Toxoplasma*-specific event since the transcription factor is not activated by the closely related protozoan parasite, *Neospora caninum*. We further demonstrate that Serum Response Factor activation requires a parasite-derived secreted factor that signals via host MAP kinases but independently of the host actin cytoskeleton. Together, these data define Serum Response Factor as a host cell transcription factor that regulates immediate early gene expression in *Toxoplasma*-infected cells.

## Introduction

*Toxoplasma gondii* is an obligate intracellular protozoan parasite that is an important pathogen of fetuses and immune-compromised patients [1], [2]. The tissue damage seen in these individuals is the direct result of the parasite's lytic growth cycle that is composed of repeated rounds of host cell invasion, replication, and egress [3]. *Toxoplasma*'s ability to complete each round of this cycle is dependent on the parasite modulating its host cell. Host cell pathways/processes targeted by the parasite include the microtubule cytoskeleton [4], [5], [6], organelle localization [7], apoptosis [8], [9], and transcription [10], [11], [12]. In most cases, the parasite factors that direct these changes as well their targets within the host cell are unknown.

We and others have used transcriptional profiling assays as an approach to identify host cell pathways important for *Toxoplasma* growth [10], [11], [12]. These studies demonstrated that infection leads to upregulation of genes at both early (within 2 hpi) and late time points following infection. While many of the early genes encode proteins involved in host immune responses, others including c-jun, junB, c-myc, EGR1, and EGR2 were also upregulated. These genes, which are well known immediate early response genes in growth factor-stimulated cells, encode transcription factors that regulate cell survival and growth genes [13], [14]. The rapid responsiveness of these genes to growth factor stimulation is due to serum response elements (SREs) in the promoters of these genes [15].

SREs are bipartite DNA-binding sites consisting of a binding site for Serum Response Factor (SRF) and an adjacent site for the Ternary Complex Factors (TCFs), which are a family of ets-domain containing transcription factors of which ELK1 and SAP1a are the two best described [16], [17]. Growth factor signaling activates SRF/TCF by virtue of Mitogen Activated Protein (MAP) kinase phosphorylation of TCF proteins [18]. SRF can also bind to a distinct promoter element found in many genes that encode actin cytoskeleton associated proteins [19]. To regulate these genes, SRF interacts with MAL and other myocardin/MKL family members [20]. These proteins are normally bound to g-actin, which prevents them from interacting with SRF. But changes in g-actin levels release MAL that allows MAL to interact with SRF and modulate gene expression [21].

The goal of this study was to determine whether parasite activation of host immediate early genes was due to *Toxoplasma* signaling through SRF. Here we report that *Toxoplasma* activates SRF through MAPK signaling. SRF activation is dependent on release of a parasite-derived secreted factor that is likely *Toxoplasma* specific since SRF was not activated by the closely related parasite *Neospora caninum*.

## Materials and Methods

### Cells and Parasites

Human foreskin fibroblasts (HFFs), HeLa cells, and murine embryonic fibroblasts (MEFs) were grown in DMEM containing 10% heat-inactivated fetal bovine serum (FBS), L-glutamine, and penicillin/streptomycin as previously described [22]. COS cells stably transfected with SRE-CD8 were grown in the same media supplemented with G418 and were kindly provided by Dr. Richard Treisman (London Research Institute) [21]. SB203580, U0126, and SP600125 were from Calbiochem (San Diego, CA). Cytochalasin D, 4-bromophenacyl bromide and Epidermal Growth Factor (EGF) were purchased from Sigma (St. Louis, MO). Recombinant full length lethal factor, lethal factor-TcdB fusion, and protective antigen were purified from *E. coli* as previously described [23] and were provided by Dr. Jimmy Ballard (Univ. of Oklahoma Health Sciences Center).

The RH (Type I), GT1 (Type I), Pru (Type II), CTG (Type III) *Toxoplasma* and *Neospora caninum* NC-1 (from Dr. Dan Howe; Univ. of Kentucky) strains were grown in HFFs [10]. RH stably expressing both β-galactosidase and GFP were provided by Dr. Gustavo Arrizabalaga (Univ. of Idaho) [4]. All parasites and host cells were routinely tested for *Mycoplasma* contamination using the MycoAlert Mycoplasma Detection Assay kit from Lonza (Basal, Switzerland) and found to be negative. Unless otherwise stated, experiments were performed at an apparent multiplicity of infection (MOI) of 10∶1 (parasites∶host cells) and parasite numbers were determined by counting with a hemocytometer. Parasites were prepared by passage through a 27-gauge needle twice to lyse host cells and then were extensively washed. Heat-killed parasites were prepared by incubating purified parasites at 50°C for 20 minutes.

### Luciferase Assays

MEFs were transfected and luciferase activity measured as previously described [24]. The SRE-luc plasmid was purchased from Stratagene (La Jolla, CA), pEGR4x-Luc was previously described [24], SM22α-luc plasmid was provided by Dr. James Tomasek (Univ. of Oklahoma Health Sciences Center) and dominant negative p38 MAPK was from Dr. Roger Davis (Univ. of Massachusetts). All cells were co-transfected with the pTK-Rel (Promega; Madison, WI) to normalize transfection efficiencies. Pharmacological inhibitors were added to host cells at the indicated concentrations 30′ before infection. Serum stimulation was achieved by incubating cells overnight in media containing 0.1% heat-inactivated FBS and then adding fresh media containing 15% FBS.

### siRNA Transfections

Fifty µl of a 100 nM solution containing equimolar amounts of 3 different siRNAs against SRF (Ambion; Austin TX, Catalog #'s 4593, 142734, and 142734) was added to 1 µl of Lipofectamine 2000 (Invitrogen) diluted to 50 µl of Optimem (Invitrogen) in each well of a 24-well plate. After 20′ at room temperature, 2*10^5^ HeLa cells in 400 µl of antibiotic-free DMEM were added to each well. The plates were incubated at 37°C for 24 h, rinsed 3 times with DMEM, and incubated for a further 24 h in 1 ml of complete media. Ambion's Negative Control siRNA, which has no homology to any known human gene, was used as a control.

### Real Time PCR

Real-time PCR (RT-PCR) assays were performed essentially as described [24]. Briefly total RNA was isolated using the Absolutely RNA Microprep Kit (Stratagene) and treated with RNase-free DNase (Ambion) to remove contaminating DNA. Total RNA was reverse transcribed into cDNA using random primers and Superscript III Reverse Transcriptase (Invitrogen). cDNAs were diluted 1∶10 and mixed with Power SYBR-Green PCR master mix (Applied Biosystems, Foster City, CA), and PCR reaction performed using an ABI 7500 Fast real-time PCR machine (Applied Biosystems). Primers to detect EGR1 and EGR2 were previously described [24]. The real time PCR primers used to detect SRF were: 5′ATCCCTGTTTCAGCAGTTCAGCTC3′ and 5′ATCATTCACTCTTGGTGCTGTGGG3′. Because ß-actin is a SRF target gene and because GAPDH is upregulated in *Toxoplasma*-infected cells [24], RT-PCR data were normalized using primers (5′GGCAGCAGCCAAAGACAAGTATCA3′ and 5′TCATTTAAGCTGTCTGCCATGCGG3′) to detect the HIF-1α prolyl hydroxylase enzyme 1 (PHD1), which we demonstrated is not upregulated by infection ([22] and data not shown). The efficiency of the PCR primers was determined to be between 80 and 120% of the theoretical exponential amplification from cDNA dilutions. The absence of genomic DNA contamination was verified by the lack of PCR products when RNA was used as the template for the PCR reactions. Experiments were performed in triplicate, and each experiment was repeated at least three independent times. Relative expression levels were determined as described [24].

### Bacterial Toxin Preparations

Recombinant full length lethal factor (LF), LF-TcdB fusion, and protective antigen (PA) were purified from *E. coli* as previously described [23], [25]. The LF or LF-TcdB were added in equimolar amounts with protective antigen as previously described [24]. LF activity was assessed by examining MEK2 cleavage by Western blotting lysates from cells treated with increasing concentrations of toxin cell lysates with antibodies against MEK2 (from Cell Signaling; Danvers, MA) and ß-actin (Ambion; Austin, TX). LF-TcdB activity was assessed by immunofluorescence staining with Alexa Fluor 568-conjugated phalloidin (Invitrogen; Carlsbad, CA) to detect f-actin as described [4].

### Flow Cytometry

COS cells stably expressing SRE-CD8 were mock-treated, treated with 1 µM cytochalasin D, or infected with RH-GFP parasites at a MOI of 1∶2 (parasites∶host cells). Cells were harvested by scraping 24 h later and stained with APC-conjugated mouse anti-human CD8 (clone OKT8) or mouse IgG2a as an isotype control (EBioscience; San Diego, CA).

## Results

### *Toxoplasma* Infection Specifically Activates SRF

**Figure 1:**
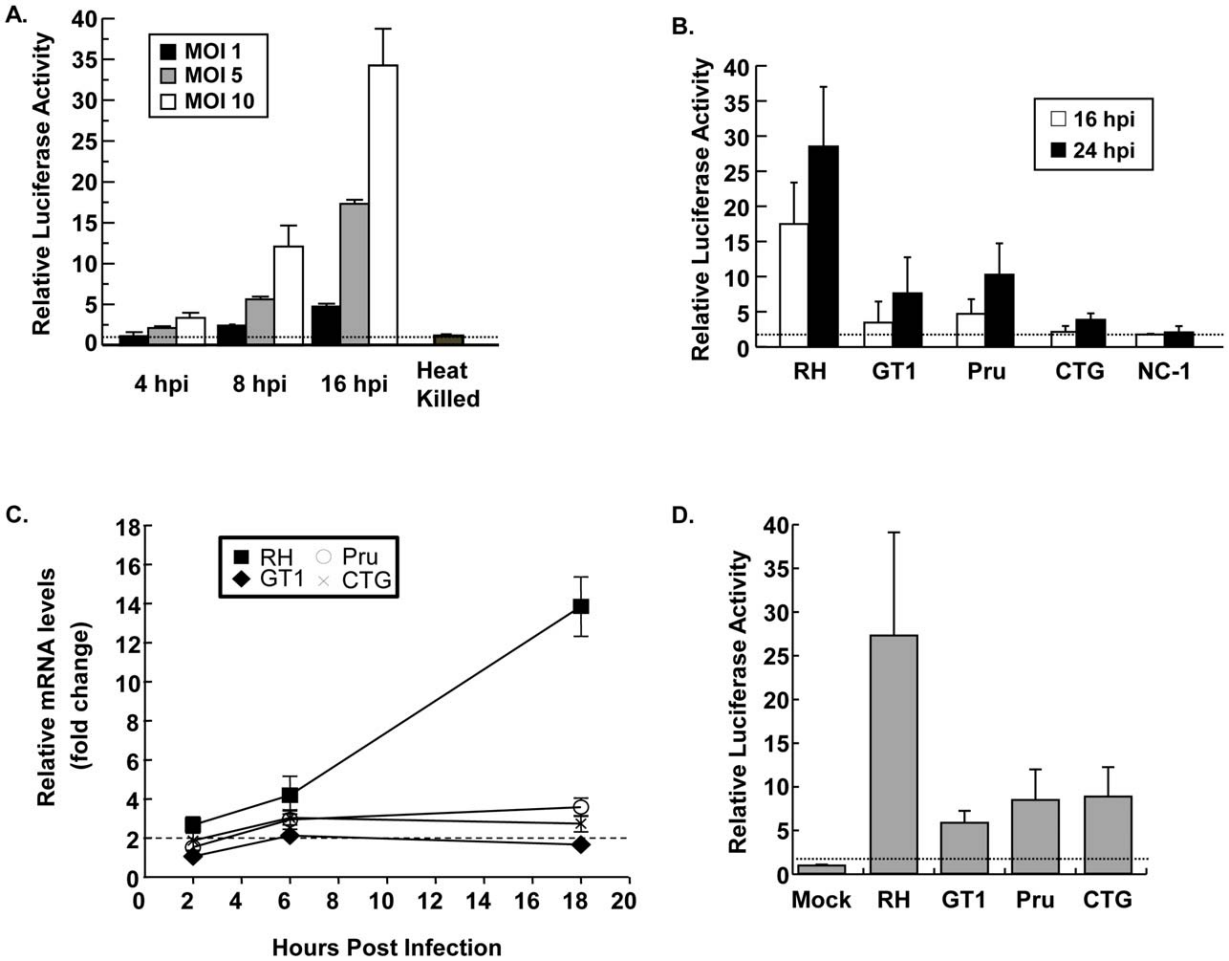
*Toxoplasma* Activates SRF. A). SRE-luc transfected MEFs were infected with increasing numbers of parasites for the indicated times and luciferase activity measured. Heat-killed parasites were added at a MOI of 10∶1 (parasites∶host cells). B). SRE-luc transfected MEFs were infected with the indicated parasites at a MOI of 10. Lysates were collected at the indicated times and luciferase activity measured. C). Cells were mock-infected or infected with equal numbers of RH and GT1 (Types I), Pru (Type II), and CTG (Type III) strains of *Toxoplasma* for indicated times before RNA was harvested. Real-time PCR was used to measure EGR2 transcript abundance. D). pEGR4x-Luc transfected cells were infected with each parasite strain and then luciferase activity was measured 16 h later. The dotted lines in all of the plots represent a 2-fold increase, which is considered the minimum increase level to be considered significant. Shown are averages and standard deviations of three independent experiments performed in triplicate.

We previously showed that *Toxoplasma* rapidly activates the host cell transcription factors EGR and AP-1 via upregulation of their mRNAs [24]. Because SRF is an important regulator of EGR and AP-1 transcription in growth-factor stimulated cells, experiments were undertaken to test whether *Toxoplasma* uses SRF to activate AP-1 and EGR. Thus, MEFs were transfected with a SRE-luc reporter in which the luciferase gene is cloned downstream of three SRF-regulated SREs. The transfected cells were then either mock-infected or infected with increasing numbers of parasites. After 4, 8, and 16 h, cells were lysed and luciferase activity was measured. The data indicated that SRE-luc activity increased in a time- and dose-dependent manner (Figure 1A) and significant increases in luciferase activity could be detected within 4 h. Incubation of heat-killed parasites (at a MOI of 10) with SRE-luc-transfected cells did not upregulate luciferase activity after 18 h indicating that SRF activation was not due to endotoxin (Fig. 1A).

Some changes in host gene expression in *Toxoplasma*-infected cells can be the result of polymorphic factors that are secreted into the host cell [12]. To determine whether SRF activation is also polymorphic, we compared SRE-luc activation in cells infected with either RH (a common laboratory type I strain) to cells infected with GT1 (Type I), Pru (Type II), or CTG (type III) strains. The cells were lysed 24 h later and luciferase activity was measured. The data indicated that SRE-luc activity was increased in cells infected with all four strains. We did note that the reporter was significantly more strongly upregulated by RH than by GT1, Pru, or CTG (Figure 1B). We also compared SRF activity by the different *Toxoplasma* strains by measuring increases in mRNA abundance of the SRF-target gene EGR2 [24]. The data indicated that similar to the SRE-luc results RH upregulated EGR2 more strongly than the other three strains (Figure 1C). Similar results were observed when we measured luciferase activity in *Toxoplasma*-infected cells transfected with an EGR luciferase reporter (Figure 1D).

To address the possibility that SRF activation was a general response of a host cell to infection, we tested whether *Neospora caninum*, which is a closely related apicomplexan parasite, also activated SRE-luc activity. In contrast to *Toxoplasma*-infected cells, SRE-luc activity remained unchanged in *Neospora*-infected cells (Fig. 1B). Together these data indicate that SRF activation is a specific response of a host cell to *Toxoplasma* and that the parasite factor that triggers SRF is most likely not polymorphic between the three major *Toxoplasma* strain types.

### SRF is Important for Immediate Early Host Gene Expression in *Toxoplasma*-Infected Host Cells

**Figure 2:**
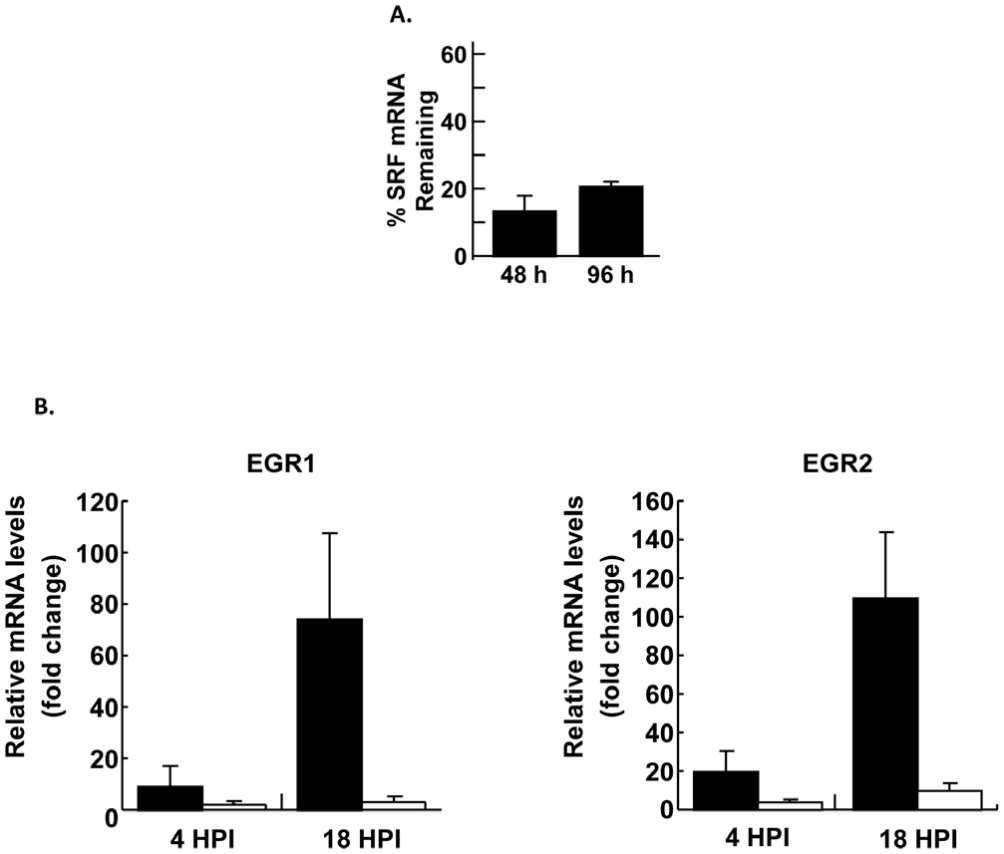
SRF is Important for EGR1 and EGR2 Induction in Parasite-Infected Cells. A). SRF mRNA abundance was measured at the indicated time points after transfection with SRF or negative control siRNAs. B). HeLa cells were transfected with SRF or negative control siRNA and infected 48 h later. RNA was collected 4 and 18 h later, converted to cDNA, and EGR1 or EGR2 transcript levels measured by real time PCR. Shown are averages and standard deviations of three independent experiments performed in triplicate.

A potential role for SRF in regulating immediate early host gene expression in *Toxoplasma*-infected cells was examined by blocking SRF expression using gene-specific siRNAs. Relative to negative control siRNAs, SRF mRNA abundance was decreased by over 80% 48 h after cells were transfected with siRNAs against SRF and this decrease lasted for at least 96 h (Figure 2A). To examine the effect of this knockdown on gene expression in *Toxoplasma*-infected cells, EGR1 and EGR2 transcript levels were measured in negative control and SRF siRNA-transfected cells 4 h and 18 h after they were mock- or parasite-infected. We found that relative to negative control siRNA-transfected cells EGR1 and EGR2 induction was reduced in SRF siRNA-transfected cells by >80% 4 hpi and >90% 18 hpi (Figure 2B). These data indicate that SRF is important for upregulating immediate early host gene expression in *Toxoplasma*-infected cells and are consistent with our earlier data showing that *de novo* protein synthesis was not required for upregulation of EGR2 mRNA 6 hpi [24].

It is possible that a reduction in EGR1 and EGR2 expression in the SRF siRNA-transfected cells was a result of decreased parasite growth. We therefore assessed parasite growth by infecting negative control and SRF siRNA-transfected cells with ß-galactosidase expressing parasites and enumerating the parasites 72 h later. The data indicated that loss of SRF had no impact on parasite growth (not shown). Similarly, loss of SRF did not affect the ability for Type II Pru strain parasites to undergo bradyzoite differentiation in the presence of the bradyzoite-inducing agent Compound 1 (not shown) [26].

### SRF Activation is Dependent on Parasite Secretion

**Figure 3:**
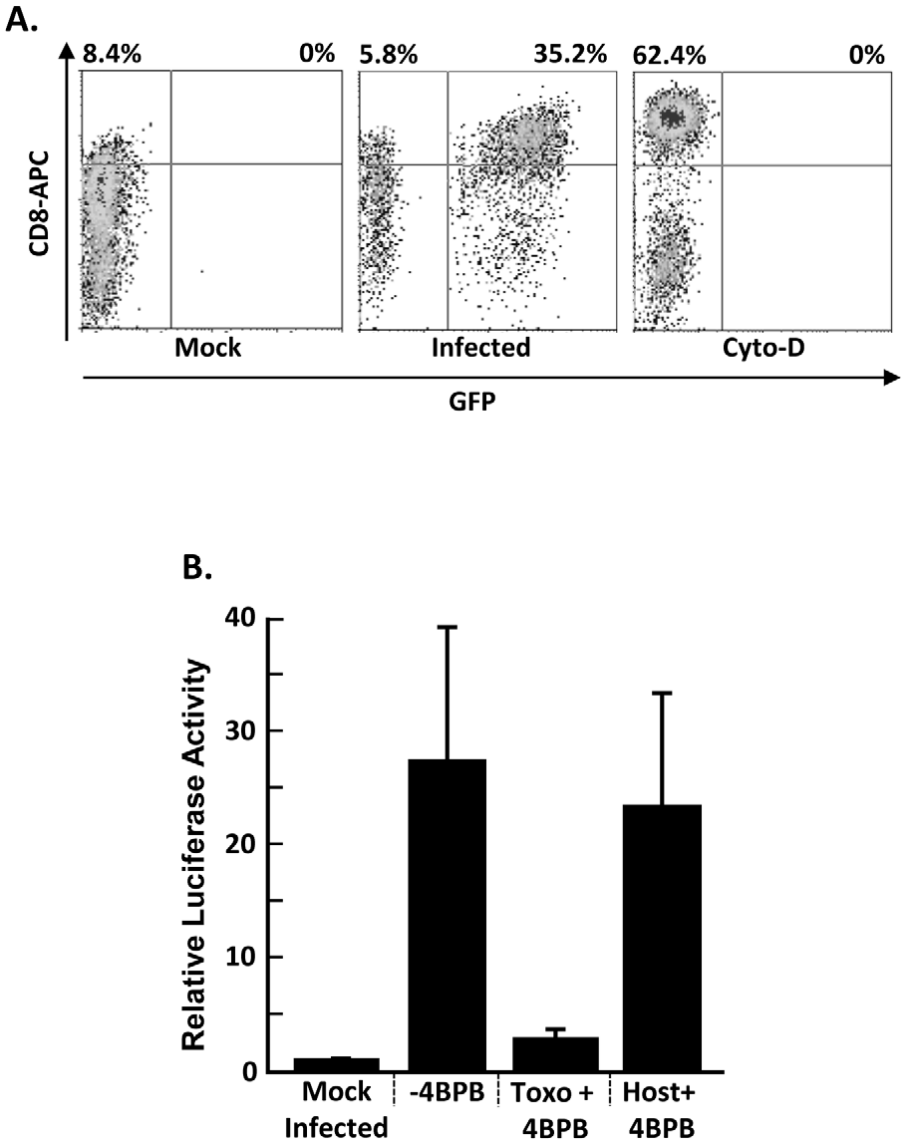
*Toxoplasma* Activation of SRF is Dependent on Rhoptry Secretion. A.) SRE-CD8 COS cells were mock treated, infected with RH GFP, or treated with 1 µM cytochalasin D. Cells were collected 18 h later and analyzed by flow cytometry after staining with anti-CD8 antibody. Shown are representative results from three independent experiments. B.) SRE-luc-transfected MEFs were mock- infected or infected with untreated parasites (−4BPB) or parasites pretreated with 4-BPB (Toxo+4BPB). In addition, SRE-luc-transfected cells were pretreated with 4-BPB and then infected with untreated parasites (Host+4BPB). Luciferase activity was measured 18 h later. Shown are the averages and standard deviations of three independent experiments performed in triplicate.

We previously demonstrated that *Toxoplasma* upregulation of EGR1 and EGR2 was mediated by a parasite-derived factor that needed to be delivered to the host cell cytoplasm [24]. We therefore hypothesized that if SRF was regulated by such a secreted factor then SRF would only be activated in infected, but not uninfected, cells. To test this hypothesis, COS cells stably transfected with human CD8 under control of SRF [19] were infected with GFP-expressing parasites or treated with cytochalasin D as a positive control [19]. The cells were harvested 18 h later, stained with anti-CD8, and analyzed by flow cytometry. As expected, cytochalasin D upregulated CD8 expression levels (Figure 3A). Similarly, CD8 expression was increased only on the surface of parasite-infected cells.

To further demonstrate a requirement for parasite secretion in activating SRF, we used 4-bromophenacyl bromide (4-BPB), which is an irreversible inhibitor of rhoptry and most likely dense granule secretion [27], which are two parasite organelles whose contents are released into the host cytosol [28], [29]. We therefore pretreated the parasites or SRE-luc transfected host cells with 1 µM 4-BPB for 15′. After washing out the drug, the treated parasites or host cells were incubated with untreated SRE-luc transfected host cells or parasites, respectively. The cells were lysed 18 h later and luciferase activity was measured. While SRE-luc activity was unaffected in the 4-BPB-treated host cells, SRE-luc activation was significantly reduced when cells were infected with pre-treated parasites (Figure 3B). Together with our previous data excluding parasite microneme- and surface-localized factors from stimulating EGR expression [24], these data indicate that SRF activation is mediated by a parasite-derived rhoptry- or dense granule-localized factor.

### *Toxoplasma* Signals Exclusively Through the SRF/TCF Pathway

**Figure 4:**
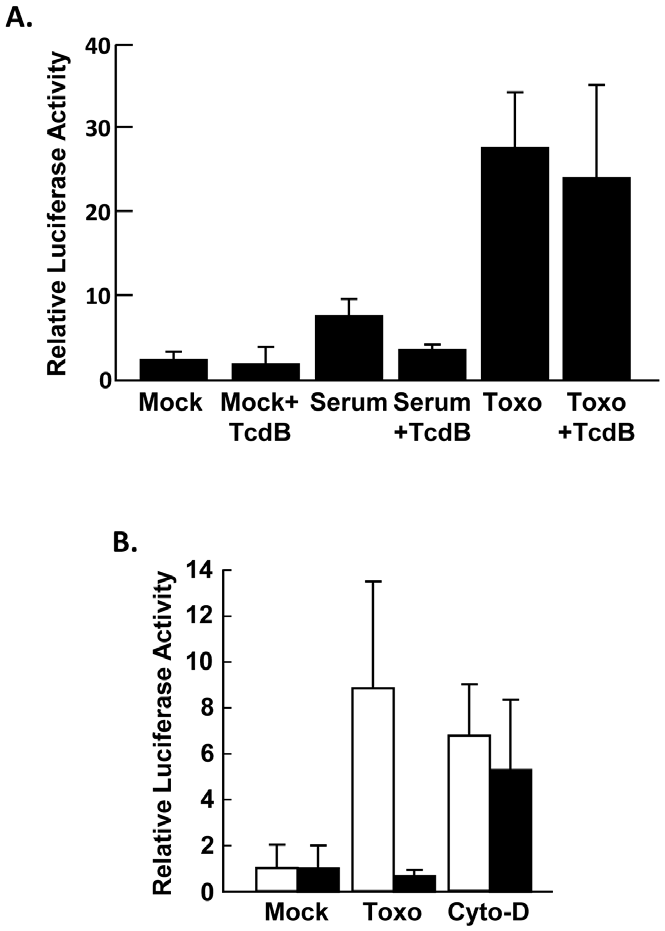
*Toxoplasma* Does Not Signal Through MAL. A.) SRE-luc transfected MEFs were incubated with TcdB-LF and PA for 6 hours. Cells were washed and either treated with 15% FBS for 6 h or infected for 16 h at which time luciferase activity was measured. B.) MEFs transfected with either SRE-luc (white bars) or pSM22α-luc (black bars) were parasite-infected or treated with 1 µM cytochalasin D. Luciferase was activity measured 16 h later. Shown are the averages and standard deviations of three independent experiments performed in triplicate.

SRF functions in concert with either MAL or the TCF complex. To test whether parasite activation of SRF is MAL-dependent, we first assessed SRE-luc activity in cells unable to activate MAL dependent transcription. Thus, SRE-luc transfected host cells were pretreated with a fusion protein in which the catalytic domain of *Clostridium difficile* TcdB toxin, is cloned downstream of the 255 amino-terminal residues of *Bacillus anthracis* LF [25]. This domain facilitates LF entry when it is incubated with *B. anthracis* PA, which is the cell binding determinant of Anthrax Toxin [30]. TcdB inhibits MAL-dependent SRF activity due to its glucosylation and inactivation of Rho GTPases that cause f-actin disassembly [31], [32], [33]. Phalloidin staining showed actin cytoskeletal disorganization in the toxin-treated cells confirming TcdB-LF fusion protein uptake and activity (not shown). We next mock-infected, parasite-infected, or serum-stimulated TcdB-LF-treated, SRE-luc-transfected cells and then measured luciferase activity. As expected, addition of TcdB significantly reduced SRF activation in serum-stimulated cells (Figure 4A). In contrast, *Toxoplasma* infection similarly increased SRE-luc activity in the absence or presence of the toxin.

SM22α is a cardiac specific protein whose promoter is activated by changes in actin cytoskeletal dynamics but not growth factor signaling since it is responsive to myocardin (a MAL family member) but not TCF [34]. To test whether *Toxoplasma* infection can stimulate MAL/myocardin-dependent SRF activity, luciferase activity was compared between host cells transfected with either the SRE-luc or SM22α-luc reporters. In contrast to cytochalasin D that stimulated luciferase activity of both reporters, parasite infection only upregulated the SRE-luc reporter (Figure 4B). These data indicate that in *Toxoplasma*-infected cells SRF activation does not require MAL, which is consistent with previous data showing that few actin-associated genes are upregulated by infection [10], [12].

### MAPK Signaling is Necessary for SRF Activation in *Toxoplasma*-Infected Cells

**Figure 5:**
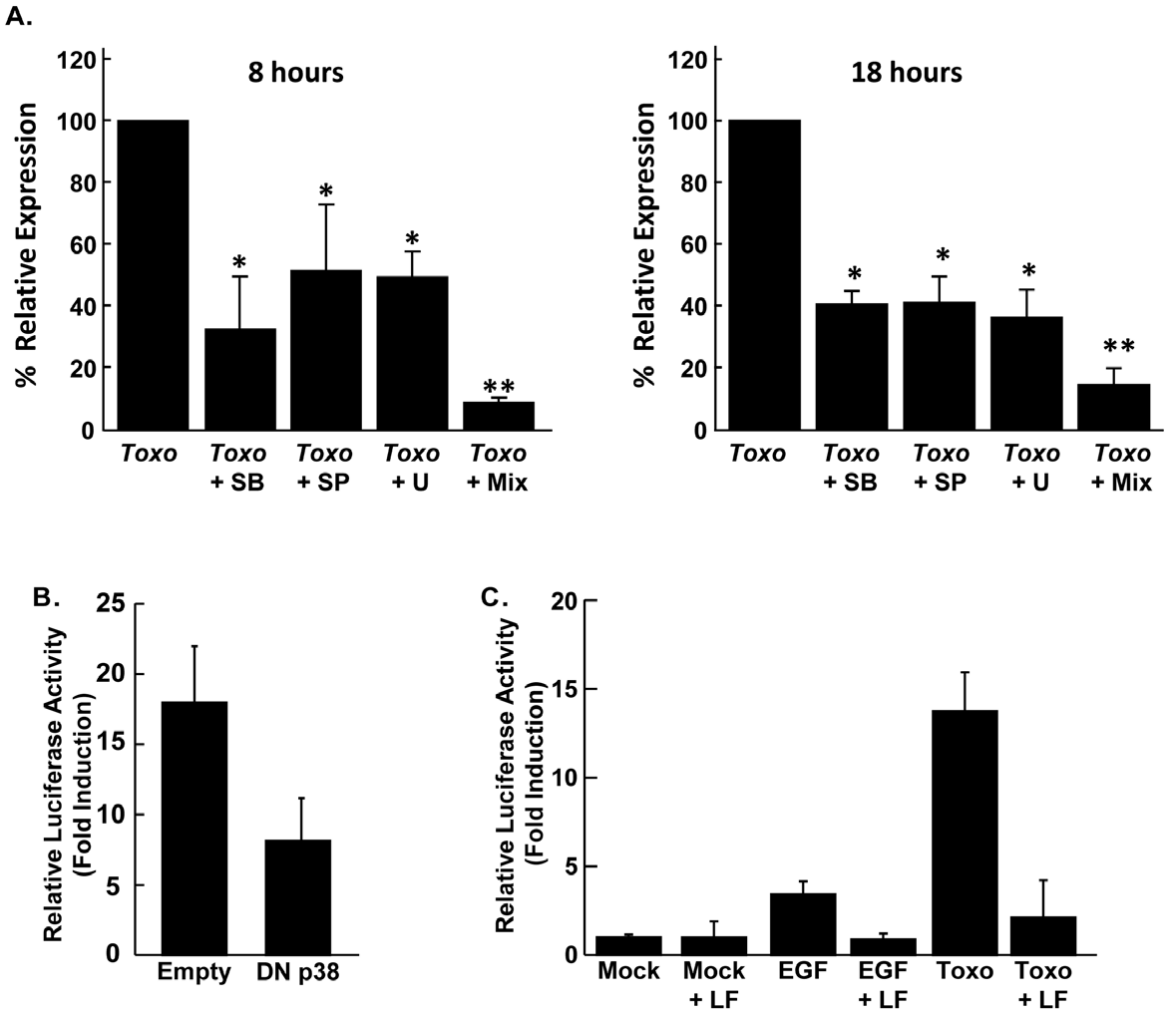
Activation of SRF by *Toxoplasma* is MAPK Dependent. A.) SRE-Luc transfected cells were mock-treated or treated for 30′ with 15 µM p38 MAPK inhibitor SB203580 (SB), 25 µM JNK inhibitor SP600125 (SP), 10 µM ERK inhibitor U0126 (U), or a mix of all three. The cells were then infected and luciferase activity measured 8 or 18 hpi. *,p<0.05 Student's *t* test, **,p<0.001 Student's *t* test. B.) SRE-luc-MEFs were co-transfected with either a control pCDNA3.1 empty vector (empty) or dominant negative p38 MAPK (DN p38). The cells were then mock- or parasite-infected and luciferase activity measured 18 h later. C.) SRE-Luc transfected cells were pretreated with LF and PA for 6 hours. Cells were washed and then either treated with EGF (50 ng/ml) for 6 hours or *Toxoplasma*-infected for 16 hours. Luciferase activity was measured at each time point. Shown are the averages and standard deviations of three independent experiments performed in triplicate.

MAPK signaling pathways are activated in parasite infected cells [35], [36], [37] and mediate TCF/SRF activation [16]. Thus, luciferase activity was measured 8 and 18 hpi in parasite-infected, SRE-luc-transfected cells that were pretreated with inhibitors of p38 (SB203580), ERK (U0126), and JNK (SP600125) MAPKs. All three inhibitors reduced SRE-luc activity in parasite-infected cells with p38 MAPK inhibition having the most significant effect at 8 hpi (Figure 5A).

The importance for p38 MAPK signaling in SRF activation in *Toxoplasma*-infected cells was assessed by co-transfecting the SRE-luc cells with either an empty plasmid or a plasmid encoding a dominant negative p38 MAPK mutant. Consistent with the pharmacological data, expression of dominant negative p38 MAPK significantly reduced SRE-luc expression by ∼60% in parasite-infected cells (Figure 5B).

We next tested the hypothesis that activation of all three major MAPK signaling modules was necessary for SRF activation in *Toxoplasma*-infected cells. First, SRE-luc activity was measured in *Toxoplasma*-infected host cells pretreated with a mixture of the three inhibitors. We found that SRE-luc activation was reduced >90% when host cells were treated with all three inhibitors (Figure 5A “*Toxo*+Mix”). Because pharmacological inhibitors can have unintended effects on the parasite as well as the host cell, host MAPK signaling was specifically inhibited with full length LF, whose catalytic domain is a metalloprotease that cleaves the MAP kinase kinases (MEKs), which are the direct upstream regulators of p38, ERK, and JNK MAPKs [38], [39], [40]. SRE-luc transfected cells were pretreated with LF and PA for 6 h, which was the minimal time needed to reduce host MEK2 to below detectable levels (not shown). The cells were then mock-infected, parasite-infected, or treated with EGF, which activates SRF in a MAPK-dependent fashion [18]. LF treatment reduced both EGF- and parasite-stimulated increases in SRE-luc activity (Figure 5C). This effect was not a consequence of using a bacterially expressed recombinant protein since the LF-TcdB fusion protein, which was similarly expressed in and purified from *E. coli*, had no effect on parasite activation of the SRE-luc (Figure 4A). Together, these data indicate that *Toxoplasma* activation of SRF/TCF is dependent on collective ERK, JNK, and p38 MAPK signaling.

## Discussion

Once a host cell comes in contact with *Toxoplasma*, numerous events take place including changes in host cell transcription [41], [42]. This is accomplished, in part, by activation of transcription factors such as HIF-1, STAT3/6, NF-κB, AP-1, and EGR. It is becoming increasing clear that *Toxoplasma* regulates these transcription factors by distinct mechanisms. As examples, HIF-1 activation is achieved by a soluble secreted factor that stimulates signaling through the Activin-Like Receptor Kinases ALK4,5,7 [22]. *Toxoplasma* regulates STAT3/6 or NF-κB by injecting into the host cell cytoplasm rhoptry- or dense granule-localized factors, respectively [43], [44], [45].

SRF activation was only detectable in parasite-infected cells and was severely reduced by an inhibitor that selectively blocks secretion of the rhoptries and most likely dense granules, but not micronemes [27]. These data are consistent with our previous work showing that upregulation of EGR2 mRNA and subsequent EGR transcription factor activity was most likely due to a rhoptry-derived factor [24]. Based on these collective data, we propose that SRF is activated by a rhoptry-derived factor although we cannot exclude the possibility of a dense granule-localized factor. The identity of this factor is not known but we believe that it is distinct from the polymorphic rhoptry kinases, ROP16 and ROP18, since activation of SRF target genes did not map to these factors [12]. Although GT1 upregulated EGR2 mRNA and the EGR luciferase reporter more weakly than the other two strains, its upregulation of SRE-luc was similar to Pru strain parasites. The basis for these differences is unclear but most likely suggests that the SRF-inducing factor is most likely not polymorphic. This conclusion is supported by microarray comparisons of strain-dependent differences in host gene expression [12].

We also observed that SRE-luc was more strongly activated by type I RH parasites than by GT1 type I parasites. Although the sequences of these two strains are highly similar, differences do exist that culminate in RH being able to survive extracellularly for longer amounts of time [46], invades more rapidly (our unpublished results), and has a significantly shorter doubling time [46]. The basis for these phenotypes is unknown but gene expression differences between the two strains are likely to be an underlying factor. As an example, ROP38 is a rhoptry-derived kinase that is secreted into host cells and down regulates the expression of several SRF target genes including EGR2. While ROP38 is abundantly expressed by GT1, Pru, and CTG strains, its expression in RH is significantly lower [47]. Thus, we propose a model in which parasite activation of host MAPK signaling leads to SRF activation and that SRF activity will be sustained in strains (e.g. RH) that express low ROP38 levels. However, it is also possible that the SRF inducing factor itself is different between RH and the three other strains and our future experiments will address this.

In some instances SRF activation is important for cell growth and survival [48], [49]. This feature led us to test whether SRF activation was important for *Toxoplasma* replication within its host cell and we found that parasite growth was not dependent on SRF. In addition, we could not detect a role for SRF in bradyzoite development after the parasites were treated with the bradyzoite-inducing agent, Compound 1 [26]. Thus, under the conditions of these *in vitro* assays SRF activation appears to be dispensable. But whether SRF is important for parasite growth in other cell types and/or growth conditions or if it has a role in virulence will be the focus of future experiments.
